# Study on the expression of lactate transport-related proteins in follicular lymphoma and their relationship with clinicopathologic features

**DOI:** 10.3389/fcell.2026.1697031

**Published:** 2026-04-13

**Authors:** Zheng Xu, Xuemei Wang, Jiuling Li, Xin Zhang, Xueju Wang

**Affiliations:** Department of Pathology, China-Japan Union Hospital, Jilin University, Changchun, Jilin, China

**Keywords:** CD147, clinicopathologic features, follicular lymphoma, MCT1, MCT4

## Abstract

**Objective:**

To investigate the expression of the lactate transport-related proteins MCT1, MCT4, and their chaperone CD147 in follicular lymphoma (FL), and to delineate their potential correlations with clinicopathological parameters predictive of prognosis.

**Methods:**

Immunohistochemistry was employed to evaluate the expression of MCT1, MCT4, and CD147 in tumor cells. Associations between protein expression and clinicopathological variables relevant to patient outcome were statistically analyzed.

**Results:**

1. MCT1-positive immunoreactivity was significantly associated with elevated serum β2-microglobulin (β2-M, a small circulating protein that rises with disease activity and serves as a marker of tumor load and adverse outcomes in lymphomas) level, male sex, and Ki-67 ≥ 30%; MCT4-positive immunoreactivity was significantly associated with Ki-67 ≥ 30%; CD147-positive immunoreactivity correlated significantly with low-grade FL. 2. Four distinct expression patterns of MCT1/MCT4 were identified: double-positive, double-negative, MCT1-positive only, and MCT4-positive only. High-grade FL demonstrated a significant predilection for either double-positive or single-positive expression; Ki-67 < 30% was significantly linked to the double-negative expression; elevated serum β2-M was significantly associated with MCT1-positive only expression; age ≥60 years and female sex were significantly associated with MCT4-positive only expression.

**Conclusion:**

The expression of MCT1, MCT4, and CD147 in FL is correlated with adverse clinicopathological features, thereby furnishing additional evidence for refinement of the Follicular Lymphoma International Prognostic Index (FLIPI, a five-parameter clinical score predicting outcome in newly diagnosed follicular lymphoma). Targeted inhibition of MCT1 and MCT4 may represent a novel therapeutic strategy. Furthermore, the heterogeneous expression of these three proteins suggests metabolic heterogeneity within FL, offering a mechanistic basis for future investigations.

## 1 Introduction

FL, which originates from germinal-center B cells, represents the second most common subtype of non-Hodgkin lymphoma in adults (Calvo et al., 2008). Although its prototypical biological behavior is indolent, the clinical outcome is markedly heterogeneous: whereas some patients succumb to rapid disease progression, others experience prolonged survival exceeding 2 decades (Horn et al., 2022). This heterogeneity remains a central obstacle to precision medicine in FL. Emerging evidence indicates that monocarboxylate transporter (MCT)–mediated lactate flux is indispensable for maintaining neoplastic energy homeostasis and for conferring resistance to microenvironmental acidification. MCT1 primarily orchestrates lactate uptake, thereby sustaining aerobic glycolysis and ATP generation, whereas MCT4 predominantly facilitates lactate extrusion, preventing intracellular lactate accumulation and its attendant cytotoxicity (Duan et al., 2022). CD147, a chaperone glycoprotein, ensures the correct membrane localization of both MCT1 and MCT4 and is therefore essential for their transporter activity (Kirk et al., 2000). The expression or overexpression of MCT1, MCT4, and CD147 is significantly associated with poor prognosis in various types of tumors (Landras et al., 2019; Ufuk et al., 2022); however, a systematic investigation of the expression of these lactate transport-related proteins in FL, as well as their correlation with clinicopathological variables, is currently lacking.

This study aims to clarify the expression of MCT1, MCT4, and CD147 in FL tumor cells and to explore whether their expression correlates with clinicopathological features associated with prognosis, thereby providing novel insights and a rational basis for diagnostic and therapeutic advances in FL.

## 2 Materials and methods

### 2.1 Materials

#### 2.1.1 Case selection

Consecutive 98 patients with newly diagnosed FL at the Department of Pathology, China–Japan Union Hospital of Jilin University, between 4 July 2017 and 30 June 2024 were screened. Inclusion criteria: (i) histopathological confirmation of FL in-house and (ii) availability of sufficient formalin-fixed paraffin-embedded (FFPE) tissue. Exclusion criteria: (i) consultation slides from external institutions, (ii) any prior lymphoma-directed therapy, (iii) concurrent or previous non-lymphomatous malignancies, (iv) renal insufficiency, (v) autoimmune diseases, (vi) active infectious diseases, and (vii) other hematological disorders. Due to limitations of the available FFPE tissue, a total of 96 samples were included in the CD147 analysis. This study was approved by the ethics review committee of China-Japan Union Hospital, Jilin University (approval number: 2026032602).

#### 2.1.2 Reagents

Primary monoclonal antibodies: mouse anti-human MCT1, mouse anti-human MCT4, and mouse anti-human CD147 (all from Santa Cruz Biotechnology, Dallas, TX, United States). Working dilutions were 1:500 for MCT1, 1:100 for MCT4, and 1:800 for CD147.

### 2.2 Methods

#### 2.2.1 Immunohistochemistry

FFPE tissue blocks were sectioned at 3 μm, mounted on charged slides, and dried at 65 °C for 30 min to ensure adhesion. After deparaffinization and rehydration, antigen retrieval was performed using a high-pH EDTA buffer in a pressure cooker. Endogenous peroxidase activity was quenched with 3% hydrogen peroxide. Slides were incubated overnight at 4 °C with appropriately diluted primary antibodies. Detection was completed using a horseradish peroxidase-conjugated secondary antibody followed by DAB chromogen. Sections were counterstained with hematoxylin, dehydrated, cleared, and coverslipped.

#### 2.2.2 Scoring and interpretation

Three experienced hematopathologists independently evaluated all immunostained slides under double-blind conditions. The percentage of neoplastic cells exhibiting membranous and/or cytoplasmic staining was recorded for each marker and scored as follows: 0 (<5%), 1 (5–<25%), 2 (25%–50%), or 3 (>50%). Scores of 0–1 were classified as negative, whereas scores of 2–3 were classified as positive (Cao et al., 2018). Results for any additional immunohistochemical stains were retrieved from the original pathology reports.

#### 2.2.3 Statistical analysis

Data were analyzed using IBM SPSS Statistics for Windows, version 25.0 (IBM Corp., Armonk, NY, United States). Categorical variables were presented as frequencies and percentages, and group comparisons were performed with the χ^2^ test or Fisher’s exact test as appropriate. P-value <0.05 was considered statistically significant.

## 3 Results

### 3.1 Clinicopathological characteristics

The overall positive rates for MCT1, MCT4, and CD147 expression were 70.4%, 77.6%, and 71.9%, respectively. The cohort comprised 43 men and 55 women. The 98 enrolled patients had a median age of 55 years (range, 27–83). 58 cases were <60 years old and 40 cases were ≥60 years. Serum β2-M level was available in 40 cases: 28 cases were within the institutional reference range (604–2,286 μg/L) and 12 cases were elevated. Serum lactate dehydrogenase (LDH, a enzyme that rises with tumor burden and predicts worse survival) level was documented in 58 cases: 39 cases were normal and 19 cases were above the upper reference limit (80–285 U/L). Among the 55 cases with measurable nodal disease, the largest lymph-node diameter was <6 cm in 48 cases and ≥6 cm in 7 cases. Hemoglobin level data were retrievable for 60 cases: 48 cases had levels ≥120 g/L and 12 cases had levels <120 g/L (local normal range: 120–160 g/L for men, 110–150 g/L for women). Histologic grading was high-grade in 40 cases, low-grade in 35 cases, and mixed-grade in 23 cases. Ki-67 index was evaluable in 98 cases: 32 cases had Ki67 < 30% and 66 cases had Ki67 ≥ 30%. p53 status was evaluable in 59 cases, revealing 18 mutant and 41 wild-type patterns. C-myc expression was quantifiable in 72 cases: 54 cases had 0 ≤ C-myc <30%, 16 cases had 30% ≤ C-myc <70%, and 2 cases had C-myc ≥70%. According to the 4th edition of the WHO classification, FL must be mandatorily graded by counting the mean number of centroblasts in ten consecutive high-power fields: ≤5 centroblasts corresponds to grade 1, 6–15 to grade 2, and >15 to grade 3, which was further subdivided into grade 3A (residual centrocytes present) and grade 3B (solid sheets of centroblasts); grades 1–2 were categorized as low-grade, whereas grade 3 was considered high-grade. Tumor grading in this study was performed in strict accordance with these WHO criteria. In addition, the reference standards for serum β2-M level, serum LDH level, and hemoglobin level were internal standards within the institution, and they were not universal standards.

### 3.2 Association of MCT1, MCT4, and CD147 expression with clinicopathological parameters in FL tumor cells

Statistical analyses revealed that MCT1-positive immunoreactivity was significantly associated with elevated serum β2-M level, male sex, and Ki-67 ≥ 30%, whereas no significant associations were observed with age, serum LDH level, nodal diameter, hemoglobin level, tumor grade, p53 status, or C-myc expression. MCT4-positive immunoreactivity demonstrated a significant correlation exclusively with Ki-67 ≥ 30%; all other clinicopathological variables showed no significant relationship with MCT4 expression. Similarly, CD147-positive immunoreactivity was significantly linked only to low-grade FL and exhibited no significant associations with any remaining parameters. Table 1 presents the comparison of clinicopathological characteristics with different MCT1 expression. Table 2 presents the comparison of clinicopathological characteristics with different MCT4 expression. Table 3 presents the comparison of clinicopathological characteristics with different CD147 expression.

**TABLE 1:** Comparison of clinicopathological characteristics with different MCT1 expression, n (%).

Indicators	MCT1 (+)	MCT1 (−)	*χ* ^2^/*χ* ^*2*^_*corrected*_/*G* ^*2*^ value	P value
Age	​	​	0.142	0.706
<60 years old	40 (58.0)	18 (62.1)	​	​
≥60 years old	29 (42.0)	11 (37.9)	​	​
Serum β2-M level	​	​	6.273	**0.012**
normal	15 (55.6)	13 (100.0)	​	​
increased	12 (44.4)	0 (0.0)	​	​
Serum LDH level	​	​	1.315	0.251
normal	25 (62.5)	14 (77.8)	​	​
increased	15 (37.5)	4 (22.2)	​	​
Lymph node diameter	​	​	**/**	0.416
≤6 cm	32 (84.2)	16 (94.1)	​	​
>6 cm	6 (15.8)	1 (5.9)	​	​
Hemoglobin level	​	​	<0.001	1.000
≥120 g/L	33 (80.5)	15 (78.9)	​	​
<120 g/L	8 (19.5)	4 (21.1)	​	​
Gender	​	​	6.517	**0.011**
female	33 (47.8)	22 (75.9)	​	​
male	36 (52.2)	7 (24.1)	​	​
Tumor grade	​	​	4.691	0.096
low-grade	24 (34.8)	16 (55.2)	​	​
high-grade	29 (42.0)	6 (20.7)	​	​
mixed-grade	16 (23.2)	7 (24.1)	​	​
Ki67	​	​	12.630	**<0.001**
<30%	15 (21.7)	17 (58.6)	​	​
≥30%	54 (78.3)	12 (41.4)	​	​
p53	​	​	<0.001	1.000
mutant	14 (29.8)	4 (33.3)	​	​
wild-type	33 (70.2)	8 (66.7)	​	​
C-myc	​	​	1.295	0.523
0≤C-myc<30%	39 (73.6)	15 (78.9)	​	​
30%≤C-myc<70%	12 (22.6)	4 (21.1)	​	​
C-myc≥70%	2 (3.8)	0 (0)	​	​

Bold font indicates statistical significance.

**TABLE 2:** Comparison of clinicopathological characteristics with different MCT4 expression, n (%).

Indicators	MCT4 (+)	MCT4 (−)	χ^2^/χ^2^corrected value		P-value
Age	​	​	3.843		0.050
<60 years old	41 (53.9)	17 (77.3)	​		​
≥60 years old	35 (46.1)	5 (22.7)	​		​
Serum β2-M level	​	​	0.159		0.690
normal	20 (66.7)	8 (80.0)	​		​
increased	10 (33.3)	2 (20.0)	​		​
Serum LDH level	​	​	0.259		0.611
normal	29 (64.4)	10 (76.9)	​		​
increased	16 (35.6)	3 (23.1)	​		​
Lymph node diameter	​	​	0.069		0.794
≤6 cm	35 (85.4)	13 (92.9)	​		​
>6 cm	6 (14.6)	1 (7.1)	​		​
Hemoglobin level	​	​	0.052		0.819
≥120 g/L	36 (78.3)	12 (85.7)	​		​
<120 g/L	10 (21.7)	2 (14.3)	​		​
Gender	​	​	0.102		0.750
female	42 (55.3)	13 (59.1)	​		​
male	34 (44.7)	9 (40.9)	​		​
Tumor grade	​	​	4.769		0.092
low-grade	27 (35.5)	13 (59.1)	​		​
high-grade	31 (40.8)	4 (18.2)	​		​
mixed-grade	18 (23.7)	5 (22.7)	​		​
Ki67	​	​	9.017		**0.003**
<30%	19 (25.0)	13 (59.1)	​		​
≥30%	57 (75.0)	9 (40.9)	​		​
p53	​	​	/		0.184
mutant	14 (26.9)	4 (57.1)	​		​
wild-type	38 (73.1)	3 (42.9)	​		​
C-myc	​	​	0.899		0.638
0≤C-myc<30%	43 (74.1)	11 (78.6)	​		​
30%≤C-myc<70%	13 (22.4)	3 (21.4)	​		​
C-myc≥70%	2 (3.4)	0 (0)	​		​

Bold font indicates statistical significance.

**TABLE 3:** Comparison of clinicopathological characteristics with different CD147 expression, n (%).

Indicators	CD147 (+)	CD147 (−)	*χ* ^2^/*χ* ^*2*^_*corrected*_ value	*P* value
Age	​	​	1.963	0.161
<60 years old	44 (63.8)	13 (48.1)	​	​
≥60 years old	25 (36.2)	14 (51.9)	​	​
Serum β2-M level	​	​	1.847	0.174
normal	15 (60.0)	8 (100.0)	​	​
increased	10 (40.0)	0 (0)	​	​
Serum LDH level	​	​	0.174	0.677
normal	28 (68.3)	10 (62.5)	​	​
increased	13 (31.7)	6 (37.5)	​	​
Lymph node diameter	​	​	0.085	0.771
≤6 cm	34 (85.0)	13 (92.9)	​	​
>6 cm	6 (15.0)	1 (7.1)	​	​
Hemoglobin level	​	​	2.127	0.145
≥120 g/L	34 (85.0)	11 (64.7)	​	​
<120 g/L	6 (15.0)	6 (35.3)	​	​
Gender	​	​	0.295	0.587
female	40 (58.0)	14 (51.9)	​	​
male	29 (42.0)	13 (48.1)	​	​
Tumor grade	​	​	7.481	**0.024**
low-grade	33 (47.8)	6 (22.2)	​	​
high-grade	19 (27.5)	15 (55.6)	​	​
mixed-grade	17 (24.6)	6 (22.2)	​	​
Ki67	​	​	0.928	0.336
<30%	25 (36.2)	7 (25.9)	​	​
≥30%	44 (63.8)	20 (74.1)	​	​
p53	​	​	0.445	0.505
mutant	11 (28.2)	7 (36.8)	​	​
wild-type	28 (71.8)	12 (63.2)	​	​
C-myc	​	​	0.511	0.775
0≤C-myc<30%	37 (74.0)	15 (75.0)	​	​
30%≤C-myc<70%	12 (24.0)	4 (20.0)	​	​
C-myc≥70%	1 (2.0)	1 (5.0)	​	​

Bold font indicates statistical significance.

### 3.3 Expression patterns of MCT1/MCT4 in FL tumor cells

**FIGURE 1:**
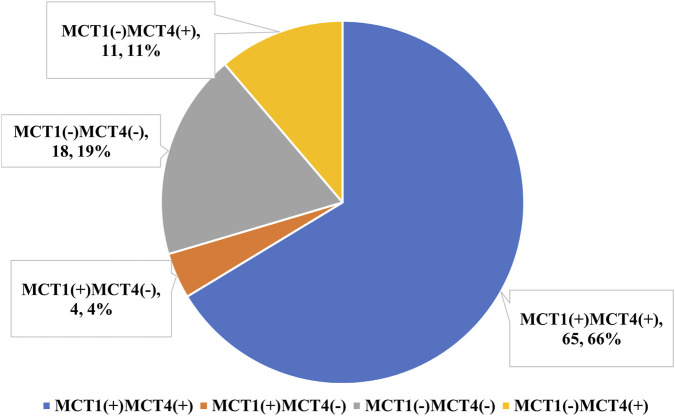
Distribution of expression patterns of MCT1/MCT4 in FL tumor cells.

A prior breast-cancer study documented the coexistence of MCT1 and MCT4 positivity in neoplastic cells (Hong et al., 2016). As illustrated in Figure 1, four distinct expression patterns of MCT1/MCT4 were identified in the present FL cohort: double-positive, double-negative, MCT1-positive only, and MCT4-positive only. The double-positive pattern predominated, accounting for 66.33% of cases, whereas MCT1-positive only was the least frequent (4.08%). The double-negative and MCT4-positive only groups were observed in 18.37% and 11.22% of cases, respectively.

### 3.4 Clinicopathological differences across the expression patterns of MCT1/MCT4 in FL tumor cells

**FIGURE 2:**
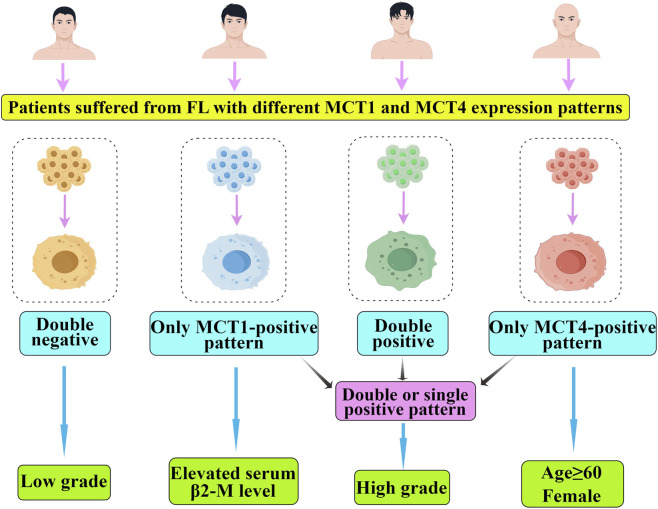
The association between the expression patterns of MCT1/MCT4 and prognostic-related clinical pathological features.

Statistical analyses revealed significant heterogeneity among the four defined expression patterns of MCT1/MCT4 with respect to several clinicopathological parameters. Age distribution showed that patients ≥60 years old were significantly enriched in the MCT4-positive only pattern. Regarding serum β2-M level, the MCT1-positive only pattern was the sole category significantly associated with elevated β2-M level. Female sex was preferentially represented within the MCT4-positive only pattern. With respect to histological grade, the distribution of expression patterns of MCT1/MCT4 differed significantly among low-grade, high-grade, and mixed-grade FL. Furthermore, Ki-67 index was markedly lower in the double-negative pattern relative to the other three patterns. No statistically significant associations were observed for serum LDH level, nodal diameter, hemoglobin level, p53 status, or C-myc expression. Tables 4-6 illustrate the clinicopathological differences across the expression patterns of MCT1/MCT4 in FL tumor cells. Figure 2 summarizes the content of this section.

**TABLE 4:** Comparison of clinicopathological characteristics among different expression patterns of MCT1/MCT4, n (%).

Indicators	MCT1 (+) MCT4 (+)	MCT1 (−) MCT4 (−)	*χ* ^2^/*χ* ^*2*^_*corrected*_/*G* ^*2*^value	*P* value
Age	​	​	3.778	0.052
<60 years old	38 (58.5)	15 (83.3)	​	​
≥60 years old	27 (41.5)	3 (16.7)	​	​
Serum β2-M level	​	​	2.893	0.089
normal	15 (60.0)	8 (100.0)	​	​
increased	10 (40.0)	0 (0.0)	​	​
Serum LDH level	​	​	0.635	0.425
normal	24 (63.2)	9 (81.8)	​	​
increased	14 (36.8)	2 (18.2)	​	​
Lymph node diameter	​	​	0.028	0.867
≤6 cm	29 (82.9)	10 (90.9)	​	​
>6 cm	6 (17.1)	1 (9.1)	​	​
Hemoglobin level	​	​	<0.001	1.000
≥120 g/L	30 (78.9)	9 (81.8)	​	​
<120 g/L	8 (21.1)	2 (18.2)	​	​
Gender	​	​	1.015	0.314
female	31 (47.7)	11 (61.1)	​	​
male	34 (52.3)	7 (38.9)	​	​
Tumor grade	​	​	9.086	**0.011**
low-grade	24 (36.9)	13 (72.2)	​	​
high-grade	26 (40.0)	1 (5.6)	​	​
mixed-grade	15 (23.1)	4 (22.2)	​	​
Ki67	​	​	15.230	**<0.001**
<30%	15 (23.1)	13 (72.2)	​	​
≥30%	50 (76.9)	5 (27.8)	​	​
p53	​	​	**/**	0.311
mutant	13 (28.9)	3 (60.0)	​	​
wild-type	32 (71.1)	2 (40.0)	​	​
C-myc	​	​	0.931	0.628
0≤C-myc<30%	37 (74.0)	9 (81.8)	​	​
30%≤C-myc<70%	11 (22.0)	2 (18.2)	​	​
C-myc≥70%	2 (4.0)	0 (0)	​	​

Bold font indicates statistical significance.

**TABLE 5:** Comparison of clinicopathological characteristics among different expression patterns of MCT1/MCT4, n (%).

Indicators	MCT1 (+) MCT4 (−)	MCT1 (−) MCT4 (−)	*G* ^*2*^ value	*P* value
Age	​	​	/	0.210
<60 years old	2 (50.0)	15 (83.3)	​	​
≥60 years old	2 (50.0)	3 (16.7)	​	​
Serum β2-M level	​	​	**/**	**0.022**
normal	0 (0.0)	8 (100.0)	​	​
increased	2 (100.0)	0 (0.0)	​	​
Serum LDH level	​	​	/	0.423
normal	1 (50.0)	9 (81.8)	​	​
increased	1 (50.0)	2 (18.2)	​	​
Lymph node diameter	​	​	/	1.000
≤6 cm	3 (100.0)	10 (90.9)	​	​
>6 cm	0 (0.0)	1 (9.1)	​	​
Hemoglobin level	​	​	/	1.000
≥120 g/L	3 (100.0)	9 (81.8)	​	​
<120 g/L	0 (0.0)	2 (18.2)	​	​
Gender	​	​	/	1.000
female	2 (50.0)	11 (61.1)	​	​
male	2 (50.0)	7 (38.9)	​	​
Tumor grade	​	​	11.359	**0.003**
low-grade	0 (0)	13 (72.2)	​	​
high-grade	3 (75.0)	1 (5.6)	​	​
mixed-grade	1 (25.0)	4 (22.2)	​	​
Ki67	​	​	**/**	**0.017**
<30%	0 (0.0)	13 (72.2)	​	​
≥30%	4 (100.0)	5 (27.8)	​	​
p53	​	​	**/**	1.000
mutant	1 (50.0)	3 (60.0)	​	​
wild-type	1 (50.0)	2 (40.0)	​	​
C-myc	​	​	**/**	1.000
0≤C-myc<30%	2 (66.7)	9 (81.8)	​	​
30%≤C-myc<70%	1 (33.3)	2 (18.2)	​	​

Bold font indicates statistical significance.

**TABLE 6:** Comparison of clinicopathological characteristics among different expression patterns of MCT1/MCT4, n (%).

Indicators	MCT1 (−) MCT4 (+)	MCT1 (−) MCT4 (−)	*G* ^*2*^/*χ* ^*2*^_*corrected*_ value	*P* value
Age			6.888	**0.009**
<60 years old	3 (27.3)	15 (83.3)		
≥60 years old	8 (72.7)	3 (16.7)		
Serum LDH level			/	1.000
normal	5 (71.4)	9 (81.8)		
increased	2 (28.6)	2 (18.2)		
Lymph node diameter			/	1.000
≤6 cm	6 (100.0)	10 (90.9)		
>6 cm	0 (0.0)	1 (9.1)		
Hemoglobin level			/	1.000
≥120 g/L	6 (75.0)	9 (81.8)		
<120 g/L	2 (25.0)	2 (18.2)		
Gender			/	**0.026**
female	11 (100.0)	11 (61.1)		
male	0 (0.0)	7 (38.9)		
Ki67			2.292	0.130
<30%	4 (36.4)	13 (72.2)		
≥30%	7 (63.6)	5 (27.8)		
Tumor grade				
low-grade	3 (27.3)	13 (72.2)	8.086	**0.018**
high-grade	5 (45.5)	1 (5.6)		
mixed-grade	3 (27.3)	4 (22.2)		
p53			**/**	0.222
mutant	1 (14.3)	3 (60.0)		
wild-type	6 (85.7)	2 (40.0)		
C-myc			**/**	1.000
0≤C-myc<30%	6 (75.0)	9 (81.8)		
30%≤C-myc<70%	2 (25.0)	2 (18.2)		

Bold font indicates statistical significance.

## 4 Discussion

This study investigated the expression of MCT1,MCT4, and their chaperone CD147 in FL tumor cells and correlated these findings with clinicopathological variables, thereby offering novel insights into the biological behavior and prognosis of FL.

MCT1-positive immunoreactivity was significantly associated with elevated serum β2-M level, male sex and Ki67 ≥ 30%. MCT1-mediated lactate export increases extracellular lactate concentrations within the tumor microenvironment, thereby suppressing effector T-cell activity and fostering the infiltration of immunosuppressive cells (Xu et al., 2025). Lymphocytes actively secrete β2-M into the circulation, accounting for the observed rise in serum level (Igwe et al., 2022). In previous studies, elevated β2-M level has been identified as susceptibility or risk factors for diffuse large B-cell lymphoma and Hodgkin lymphoma (Li et al., 2024). Although β2-M serves as a poor prognostic indicator for FL, prior research has not established an association between elevated β2-M level and increased FL risk, likely due to confounding factors. Moreover, MCT1 promotes tumor proliferation through lactate metabolism (Xu et al., 2025), and its regulation may display sex-specific differences that warrant further investigation. MCT4-positive immunoreactivity was also significantly linked to Ki67 ≥ 30%. The concordant associations of Ki67 with both MCT1 and MCT4 underscore the central role of lactate metabolism in tumor proliferation. It should be noted that a high prevalence of MCT1 and MCT4 positivity was observed even among patients whose serum LDH levels remained within the normal range. No significant relationships were observed between MCT1, MCT4 and p53 or C-myc expression, suggesting that MCT1 and MCT4 influence FL biology primarily via metabolic and proliferative pathways rather than through apoptosis or transcriptional modulation.

CD147, the obligatory chaperone for both MCT1 and MCT4 (Bovenzi et al., 2015), was expressed in 71.9% of cases, and its positivity correlated significantly with grade. High-grade FL constituted 55.6% of the CD147-negative group, compared with only 27.5% of the CD147-positive group. CD147 was not associated with age, serum β2-M level or other systemic indices, implying that it reflects intrinsic tumor biology rather than systemic pathophysiology. The absence of correlations between CD147 and Ki67, p53 or C-myc further suggests that CD147 operates independently of canonical proliferation and apoptosis pathways. As a transmembrane glycoprotein, CD147 may facilitate tumor progression through protein–protein interactions; for instance, in mycosis fungoides and Sézary syndrome, CD147 interacts with cyclophilin A to stimulate tumor cell proliferation in autocrine and paracrine fashions (Sakamoto et al., 2021).

The predominant co-expression pattern of MCT1 and MCT4 indicates potential synergism. Functioning as bidirectional lactate transporters—MCT1 for import and MCT4 for export (Xu et al., 2025)—their concomitant expression suggests a pronounced reliance on lactate shuttling. Beyond its role as a glycolytic by-product (Jin et al., 2023), lactate activates HIF-1α-dependent angiogenic signalling within the tumor microenvironment (Wang et al., 2021). Double-positive pattern has previously been reported in breast cancer (Hong et al., 2016); its presence in FL supports the universality of lactate shuttling in tumor progression. The lowest frequency was observed in the MCT1-positive only pattern, implying that exclusive MCT1 expression is insufficient to sustain metabolic demand and requires complementary transporters.

The expression patterns of MCT1/MCT4 were significantly associated with age, serum β2-M level, sex and tumor grade. The MCT4-positive only group exhibited a higher proportion of patients aged ≥60 years and was exclusively female, suggesting that elderly women may preferentially exploit MCT4-mediated lactate export to maintain metabolic homeostasis. Age-related alterations in the stromal and immune microenvironment may drive progression from indolent to aggressive disease (Fane and Weeraratna, 2020), the latter displaying heightened metabolic activity (Huang et al., 2023) To preserve intracellular pH balance, tumor cells preferentially utilize MCT4 for lactate extrusion (Xu et al., 2025). The MCT1-positive only group showed a higher incidence of elevated serum β2-M level, as discussed above. The association between the expression patterns of MCT1/MCT4 and FL grade underscores their potential as biomarkers for tumor biology and risk stratification.

Notably, no significant correlations were observed with serum LDH level, maximum nodal diameter or haemoglobin level. Serum LDH level reflects systemic rather than local lactate efflux (Lin et al., 2021); nodal size, although prognostically relevant (Bhardwaj et al., 2022), does not linearly correlate with glycolytic flux, and haemoglobin is influenced by multiple confounders that may mask lactate-mediated effects on erythrocytes. The relative independence of expression patterns of MCT1/MCT4 from systemic indices suggests that these transporters regulate local metabolism without substantially perturbing systemic physiology, a property that may be advantageous for targeted therapy.

Ki67, a surrogate marker of proliferation, was significantly associated with expression patterns of MCT1/MCT4: 72.2% of the double-negative group exhibited Ki67 < 30%, whereas 76.9% of the double-positive group displayed Ki67 ≥ 30%. These data corroborate the direct link between metabolic reprogramming and proliferation (Sheng et al., 2023); dual inhibition of MCT1 and MCT4 disrupts lactate shuttling and thereby suppresses tumor growth. The double-positive pattern may foster metabolic symbiosis that accelerates proliferation (Sonveaux et al., 2012).

No significant associations were detected between expression patterns of MCT1/MCT4 and p53 or C-myc. Although prior studies have reported that p53 and C-myc can transcriptionally regulate MCT1 (Bo et al., 2012; Gan et al., 2016), such relationships were not evident here, suggesting either alternative regulatory mechanisms or compensatory transcription factor activity (e.g., HIF-1α) in FL.

This study also has several limitations: as FL is an indolent disease, prolonged follow-up is required to obtain mature survival data, precluding any survival analysis; the findings derive from a single-institution cohort, potentially limiting generalizability; the sample size is modest; and no independent molecular validation is performed.

In summary, the expression of MCT1, MCT4 and CD147 in FL tumor cells provide new perspectives on prognosis and therapeutic targeting. While their prognostic value remains to be definitively established, their roles in metabolic regulation highlight their potential as therapeutic targets. Integration of multi-dimensional clinical and basic research data may ultimately enable precise subclassification and individualised treatment of FL.

## Data Availability

The original contributions presented in the study are included in the article/supplementary material, further inquiries can be directed to the corresponding author.

## References

[B1] Bhardwaj D. Dasgupta A. DiCenzo D. Brade S. Fatima K. Quiaoit K. (2022). Early changes in quantitative ultrasound imaging parameters during neoadjuvant chemotherapy to predict recurrence in patients with locally advanced breast cancer. Cancers 14 (5), 1247. 10.3390/cancers14051247 35267555 PMC8909335

[B2] Boidot R. Végran F. Meulle A. Le Breton A. Dessy C. Sonveaux P. (2012). Regulation of monocarboxylate transporter MCT1 expression by p53 mediates inward and outward lactate fluxes in tumors. Cancer Res. 72 (4), 939–948. 10.1158/0008-5472.CAN-11-2474 22184616

[B3] Bovenzi C. D. Hamilton J. Tassone P. Johnson J. Cognetti D. M. Luginbuhl A. (2015). Prognostic indications of elevated MCT4 and CD147 across cancer types: a meta-analysis. BioMed Res. Int. 2015, 242437. 10.1155/2015/242437 26779534 PMC4686628

[B4] Calvo K. R. Dabir B. Kovach A. Devor C. Bandle R. Bond A. (2008). IL-4 protein expression and basal activation of erk *in vivo* in follicular lymphoma. Blood 112 (9), 3818–3826. 10.1182/blood-2008-02-138933 18682601 PMC2954752

[B5] Cao Y. W. Liu Y. Dong Z. Guo L. Kang E. H. Wang Y. H. (2018). Monocarboxylate transporters MCT1 and MCT4 are independent prognostic biomarkers for the survival of patients with clear cell renal cell carcinoma and those receiving therapy targeting angiogenesis. Urol. Oncol. 36 (6), 311.e15–311.e25. 10.1016/j.urolonc.2018.03.014 29657088

[B6] Duan Q. Zhang S. Wang Y. Lu D. Sun Y. Wu Y. (2022). Proton-coupled monocarboxylate transporters in cancer: from metabolic crosstalk, immunosuppression and anti-apoptosis to clinical applications. Front. Cell Dev. Biol. 10, 1069555. 10.3389/fcell.2022.1069555 36506099 PMC9727313

[B7] Fane M. Weeraratna A. T. (2020). How the ageing microenvironment influences tumour progression. Nat. Rev. Cancer 20 (2), 89–106. 10.1038/s41568-019-0222-9 31836838 PMC7377404

[B8] Gan L. Xiu R. Ren P. Yue M. Su H. Guo G. (2016). Metabolic targeting of oncogene MYC by selective activation of the proton-coupled monocarboxylate family of transporters. Oncogene 35 (23), 3037–3048. 10.1038/onc.2015.360 26434591

[B9] Hong C. S. Graham N. A. Gu W. Espindola Camacho C. Mah V. Maresh E. L. (2016). MCT1 modulates cancer cell pyruvate export and growth of tumors that Co-express MCT1 and MCT4. Cell Rep. 14 (7), 1590–1601. 10.1016/j.celrep.2016.01.057 26876179 PMC4816454

[B10] Horn H. Jurinovic V. Leich E. Kalmbach S. Bausinger J. Staiger A. M. (2022). Molecular cytogenetic profiling reveals similarities and differences between localized nodal and systemic follicular lymphomas. HemaSphere 6 (9), e767. 10.1097/HS9.0000000000000767 35974958 PMC9371558

[B11] Huang Y. Wang M. Jiang L. Wang L. Chen L. Wang Q. (2023). Optimal clinical protocols for total-body 18F-FDG PET/CT examination under different activity administration plans. EJNMMI Physics 10 (1), 14. 10.1186/s40658-023-00533-y 36808378 PMC9938848

[B12] Igwe C. U. Ewuga E. E. Ujowundu C. O. Onyeocha I. O. Onwuliri V. A. (2022). Serum protein concentration and amino acid profile of HIV/HBV co-infected subjects on HAART in Plateau state, Nigeria. Afr. Health Sci. 22 (1), 418–430. 10.4314/ahs.v22i1.51 36032489 PMC9382465

[B13] Jin S. Yin E. Feng C. Sun Y. Yang T. Yuan H. (2023). Regulating tumor glycometabolism and the immune microenvironment by inhibiting lactate dehydrogenase with platinum(iv) complexes. Chem. Sci. 14 (31), 8327–8337. 10.1039/d3sc01874a 37564403 PMC10411615

[B14] Kirk P. Wilson M. C. Heddle C. Brown M. H. Barclay A. N. Halestrap A. P. (2000). CD147 is tightly associated with lactate transporters MCT1 and MCT4 and facilitates their cell surface expression. EMBO Journal 19 (15), 3896–3904. 10.1093/emboj/19.15.3896 10921872 PMC306613

[B15] Landras A. Reger de Moura C. Jouenne F. Lebbe C. Menashi S. Mourah S. (2019). CD147 is a promising target of tumor progression and a prognostic biomarker. Cancers 11 (11), 1803. 10.3390/cancers11111803 31744072 PMC6896083

[B16] Li J. Wu Y. Zhang X. Wang X. (2024). Causal relationship between beta-2 microglobulin and B-cell malignancies: genome-wide meta-analysis and a bidirectional two-sample Mendelian randomization study. Front. Immunol. 15, 1448476. 10.3389/fimmu.2024.1448476 39434879 PMC11491367

[B17] Lin Y. Wu Y. Zhong P. Hou B. Liu J. Chen Y. (2021). A clinical staging proposal of the disease course over time in non-severe patients with coronavirus disease 2019. Sci. Rep. 11 (1), 10681. 10.1038/s41598-021-90111-y 34021206 PMC8140110

[B18] Sakamoto M. Miyagaki T. Kamijo H. Oka T. Boki H. Takahashi-Shishido N. (2021). CD147-Cyclophilin a interactions promote proliferation and survival of cutaneous T-Cell lymphoma. Int. J. Mol. Sci. 22 (15), 7889. 10.3390/ijms22157889 34360654 PMC8346093

[B19] Sheng G. Gao Y. Wu H. Liu Y. Yang Y. (2023). Functional heterogeneity of MCT1 and MCT4 in metabolic reprogramming affects osteosarcoma growth and metastasis. J. Orthop. Surg. Res. 18 (1), 131. 10.1186/s13018-023-03623-w 36814318 PMC9948327

[B20] Sonveaux P. Copetti T. De Saedeleer C. J. Végran F. Verrax J. Kennedy K. M. (2012). Targeting the lactate transporter MCT1 in endothelial cells inhibits lactate-induced HIF-1 activation and tumor angiogenesis. PloS One 7 (3), e33418. 10.1371/journal.pone.0033418 22428047 PMC3302812

[B21] Ufuk A. Garner T. Stevens A. Latif A. (2022). Monocarboxylate transporters are involved in extracellular matrix remodelling in pancreatic ductal adenocarcinoma. Cancers 14 (5), 1298. 10.3390/cancers14051298 35267606 PMC8909080

[B22] Wang Y. Qin L. Chen W. Chen Q. Sun J. Wang G. (2021). Novel strategies to improve tumour therapy by targeting the proteins MCT1, MCT4 and LAT1. Eur. J. Med. Chem. 226, 113806. 10.1016/j.ejmech.2021.113806 34517305

[B23] Xu Z. Wang X. Cheng H. Li J. Zhang X. (2025). The role of MCT1 in tumor progression and targeted therapy: a comprehensive review. Front. Immunol. 16, 1610466. 10.3389/fimmu.2025.1610466 40612939 PMC12222217

